# Longitudinal Associations between Early Childhood Irritability and Adolescent Depression Symptoms in Autistic Children are Mediated by Peer Relationships but Not Educational Engagement

**DOI:** 10.1017/S0954579422001316

**Published:** 2023-01-26

**Authors:** Virginia Carter Leno, Nicola Wright, Andrew Pickles, Rachael Bedford, Anat Zaidman-Zait, Connor Kerns, Lonnie Zwaigenbaum, Eric Duku, Teresa Bennett, Stelios Georgiades, Isabel M. Smith, Annie Richards, Tracy Vaillancourt, Peter Szatmari, Mayada Elsabbagh

**Affiliations:** 1 Institute of Psychiatry, Psychology & Neuroscience, King’s College London, United Kingdom; 2 Department of Psychology, Manchester Metropolitan University, United Kingdom; 3 Department of Psychology, University of Bath, United Kingdom; 4 Constantine School of Education, Tel Aviv University, Israel; 5 University of British Columbia, Vancouver, Canada; 6 University of Alberta, Canada; 7 McMaster University, Hamilton, Ontario, Canada; 8 Faculty of Medicine, Dalhousie University, Halifax, Nova Scotia, Canada; 9 Autism Research Centre, IWK Health Centre, Halifax, Nova Scotia, Canada; 10 Counselling Psychology, Faculty of Education, University of Ottawa, Ontario, Canada; 11 University of Toronto, Canada; 12 Centre for Addiction and Mental Health, Canada; 13 The Hospital for Sick Children, Toronto, Ontario, Canada; 14 Montreal Neurological Institute, Azrieli Centre for Autism Research, McGill University, Montreal, Quebec, Canada

**Keywords:** autism, irritability, depression, peer relationships, educational achievement, failure model

## Abstract

In the general population, irritability is associated with later depression. Despite irritability being more prevalent in autistic children, the long-term sequelae are not well explored. We tested whether irritability in early childhood predicted depression symptoms in autistic adolescents, and whether associations could be explained by difficulties in peer relationships and lower educational engagement. Analyses tested the longitudinal associations between early childhood irritability (ages 3-5) and adolescent depression symptoms (age 14) in a prospective inception cohort of autistic children (N=390), followed from early in development shortly after they received a clinical diagnosis. Mediators were measured in mid-childhood (age 10) by a combination of measures, from which latent factors for peer relationships and educational engagement were estimated. Results showed early childhood irritability was positively associated with adolescent depression symptoms, and this association remained when adjusting for baseline depression. A significant indirect pathway through peer relationships was found, which accounted for around 13% of the association between early childhood irritability and adolescent depression, suggesting peer problems may partially mediate the association between irritability and later depression. No mediation effects were found for education engagement. Results highlight the importance of early screening and intervention for co-occurring irritability and peer problems in young autistic children.

## Introduction

Irritability is a trans-diagnostic concept defined as “inter-individual differences in proneness to anger that may reach a pathological extent” (Vidal-Ribas, Brotman, Valdivieso, Leibenluft, & Stringaris, 2016, p. 557). In typically developing children, high levels of irritability predict increased likelihood of a range of internalizing disorders later in development, with strongest effects seen for depression (Vidal-Ribas et al., 2016; Vidal-Ribas & Stringaris, 2021). Levels of irritability are higher in autistic as compared to typically developing children (Mandy, Roughan, & Skuse, 2014; Mayes, Calhoun, Murray, Ahuja, & Smith, 2011; Simonoff et al., 2012). Given the heightened prevalence of depression in autistic individuals (Pezzimenti et al., 2019), and the association of depression with decreased quality of life and increased mortality through suicide in autistic people (Oakley, Loth & Murphy, 2021), it is important to delineate the predictors of depression and their mechanisms of effect in autistic populations. The evidence of a mechanistic relationship between irritability and later depression in typically developing populations (e.g., Dougherty et al., 2013; Savage et al., 2015; Sorcher et al., 2021), suggests that heightened irritability in early childhood may be one explanatory mechanism and thus an important target of early preventative efforts in autistic youth. However, clinical and policy decision-making requires evidence of the long-term consequences of childhood irritability in autistic individuals.

In typically developing children, irritability in early childhood (~3 years of age) predicts depression and oppositional defiant disorder three years later (Dougherty et al., 2013), and psychopathology, functional impairment, physical health and antidepressant use in adolescence, above baseline psychiatric difficulties (Sorcher et al., 2021). Evidence from genetically sensitive designs (i.e., twin studies) suggests irritability is associated with later internalizing symptoms and not the reverse (Savage et al., 2015), although others report that the overlap in the two domains may be partly driven by common genetic factors (Stringaris, Zavos, Leibenluft, Maughan, & Eley, 2012a). One proposed mechanism linking irritability and later depression is the cascading effects of irritability early in development on peer relationships and academic attainment. This framework, often referred to as the *failure model*, posits that behavioural difficulties (such as those associated with irritability) in early childhood result in conflict with others, rejection, a lack of support, poor skill development and difficulties at school, which in turn lead to experiences of rejection and failure that increase the likelihood of subsequent depression (Burke, Loeber, Lahey, & Rathouz, 2005; Capaldi, 1992; Cole, Martin, & Powers, 1997). Although the focus of this paper is testing the effects of peer relationships/educational attainment on internalizing symptoms; it should be held in mind that more complex bidirectional associations are likely present, with evidence that symptom-driven pathways are also likely present, especially for negative peer experiences such as bullying (Saint-Georges & Vaillancourt, 2020; Vaillancourt et al., 2013).

There is piecemeal evidence to support this hypothesized framework in typically developing (i.e., non-autistic) populations. Irritability is positively associated with peer problems in both clinical samples enriched for anxiety and irritability (Chen et al., 2021) and population-representative samples (Stringaris & Goodman, 2009). Emotional and behaviour problems longitudinally predict lower educational achievement in general population cohorts (McLeod & Kaiser, 2004) and samples at risk of behaviour problems (Brennan, Shaw, Dishion, & Wilson, 2012). In turn, difficulties in peer problems and educational competence are associated with internalizing symptoms in the general population (Cole, 1990; Rahman et al., 2018; Vallaincourt, Brittain, McDougall, & Duku, 2013) although others find the evidence for the effect of educational competence on depressive symptoms is less robust (i.e., more variable) in selected samples of youth at risk for behaviour problems (Patterson & Stoolmiller, 1991). Combined tests of the whole model are limited. One study, using a select sample of boys at risk for conduct disorder, reported that when an index of psychosocial impairment (made up of measurement of separation from caregiver, grade retention/dropping out of school, low school grades, and being disliked by peers) was included as a covariate, longitudinal associations between behavioural difficulties and depression became non-significant, although the significance of the mediated effect was not specifically tested (Burke et al., 2005). Thirty percent of the association between childhood irritability and suicide attempts in adolescence was mediated by peer victimization in late childhood in a Canadian population-based cohort (Forte et al., 2021), and although irritability was not measured directly, others have found that peer problems and lower academic attainment mediate the link between childhood ADHD symptoms and depressive symptoms in adolescence in a UK population-based cohort (Powell et al., 2020). However, Barker and Salekin (2012) did not find a path from irritability to internalizing difficulties via peer victimization in their analysis of data from the same cohort, instead they found that peer victimization was associated with internalizing difficulties via increased irritability. In Barker and Salekin, irritability was measured relatively late in childhood (ages 8 and 10), meaning that the impact of irritability on developing peer competence might have been missed. Pulling together the evidence from both population-based cohorts and more selected sample studies, support for the proposed failure model as a developmental mechanism appears to be reasonable in typically developing children.

Despite the high levels of irritability in autistic children (Mandy et al., 2014; Mayes et al., 2011; Simonoff et al., 2012), the longer-term consequences of high levels of this phenotypic dimension in autistic children are less well understood. Irritability and internalizing symptoms were contemporaneously associated in a clinically ascertained sample of autistic children (Mandy et al., 2014) and one population-based study found 42% of the association between the symptoms of childhood neurodevelopmental difficulties and adolescent major depressive disorder could be accounted for by irritability in mid-childhood (~ 7 years; Eyre et al., 2019). With regards to the mechanisms of effect, i.e., how irritability might increase the likelihood of later emotional difficulties, there is some limited evidence that fits with the *failure model* as proposed in typically developing samples. Irritability is associated with lower social skills in autistic children and adolescents (Neuhaus, Webb, & Bernier, 2019). Higher scores on a composite measure of challenging behaviour (which included irritability) are associated with lower school engagement in autistic children (Zaidman-Zait et al., 2020). However, whether difficulties in these domains in turn increase risk of depression in adolescence is less well known. Some have reported that the quality of (parent-rated) peer relationships was unrelated to anxiety and depression in autistic children (Dovgan & Mazurek, 2018; Kelly, Garnett, Attwood, & Peterson, 2008), or that having fewer friendships was associated with less anxiety (Mazurek & Kanne, 2010). One study found that having positive friendships was associated with increased anxiety in autistic girls (O’Connor et al., 2022). However, others have found that self-reported friendship problems were associated with depression and loneliness in autistic adolescents (Whitehouse, Durkin, Jaquet, & Ziatas, 2009). Thus, it is unclear whether the mechanism proposed to underpin pathways between irritability and depression in typically developing children also applies to autistic children. Although speculative, it may be that being autistic either buffers or augments aspects of the failure model (e.g., having fewer friends/not being as engaged at school does not predict depression as strongly if you are autistic). Although there is limited literature that has explored these two opposing hypotheses, reviews note that although academic achievement often predicts quality of life in child populations, this may not be the case in autism (Burgess & Gutstein, 2007; Moss, Mandy & Howlin, 2017). Similarly, from the limited literature described above, it appears that the quality of peer relationships may not consistently predict emotional symptoms in autistic youth, although this may in part be due to difficulties in capturing the quality of peer relationships in non-autistic children.

Research is needed to both delineate the longer-term consequences of early childhood irritability in autism and identify the mechanisms of effect. Identification of early risk factors for adolescent depression, which can be intervened on, is key to providing early support which can potentially prevent the later onset of depression. Thus, it is important to understand not only the impact of irritability, but also our hypothesized mediators, peer relationship difficulties and educational engagement, as all offer potential avenues for intervention. The current study has two aims; 1) To replicate the longitudinal association between early childhood irritability and adolescent depression symptoms reported in typically developing children in a large inception cohort of autistic children who have been followed since early childhood, and 2) To test whether the association between irritability and depression can be in part accounted for by the potential impact of irritability on mid-childhood mediating variables; peer relationships and educational engagement.

## Method

### Participants

Data for the current study were drawn from the Pathways in Autism Spectrum Disorder study, a prospective longitudinal cohort of autistic children (N = 421), recruited at time of diagnosis in early childhood from five sites across Canada (all participants had received a diagnosis within 4 months of entry to the study). Inclusion criteria upon entry to the study were (a) age between 2 and 5 years, and (b) a clinical diagnosis of autism spectrum disorder < 4 months prior to enrolment. Diagnosis was confirmed upon entry to the study using DSM-IV-TR criteria and both the Autism Diagnostic Observation Schedule (ADOS; Lord et al., 2002) and the Autism Diagnostic Inventory - Revised (ADI-R; Rutter et al., 2003). Formal training sessions were conducted at baseline to ensure that all site leads met conventional reliability criteria on both diagnostic instruments (at least 80% inter-rater agreement). Children with a diagnosis of cerebral palsy or other neuromotor disorders, identified genetic or chromosomal abnormalities, or significantly impaired vision or hearing were excluded. Caregivers were required to be verbally proficient in English (or French, in Quebec). Parents provided informed consent before completing any study measures or assessments. Assessment occurred at baseline (T1; mean age 3.41 years), approximately 6 and 12 months after baseline (T2; 3.99 years and T3; 4.51 years), at age 6 (T4; mean age 6.66 years), at four time points approximately one year apart (T5 to T8; mean ages 7.77, 8.73, 9.71, and 10.76 years), and then in adolescence (T9; mean age 13.84 years). See Table 1 for sample descriptives.

#### Missing Data

In the current study, participants were included if they had any measurement of irritability between T1 and T3, resulting in 390 participants. See Table 1 for descriptive statistics on the included sample. Attrition analyses were conducted by entering all variables of interest as predictors of T1-T3 vs. T1-T3 + T9 grouping. To aid interpretability all continuous variables were z scored and odds ratios (OR) were generated. Results showed participants who had complete data at both T1-T3 + T9 did not differ from those who had complete data at only T1-T3 in terms of early childhood irritability (*p* = .80, OR = 1.05), baseline depression symptoms (*p* = .65, OR = 1.07), IQ (*p* = .06, OR = .99), autism symptoms (*p* = .39, OR = 1.07), male:female ratio (*p* = .65, OR = .85) or site (*p* = .97, OR = .80). Participants who had complete data at both T1-T3 + T9 differed from those who had complete data at only T1-T3 in terms of maternal age when the child entered the study at T1 (*p* = .001, OR = 1.10) and T1 income (*p* = .008, OR = 1.15), such that families who had complete data at T1-T3 + T9 had older mothers upon entry and higher incomes. Income and maternal age at study entry were therefore included as covariates in all analyses.

### Measures

#### Early Childhood Irritability Symptoms

Irritability symptoms were measured at T1, T2 and T3 using three items from the parent-report CBCL (1.5-5 years version) (Achenbach & Rescorla, 2001), “temper tantrums or hot temper,” “stubborn, sullen or irritable,” and “sudden changes in mood or feelings”, rated from *not true* (0), *somewhat or sometimes true* (1), or *very true/often true* (2) (as in Wiggins, Mitchell, Stringaris, & Leibenluft, 2014). Recent work suggests this metric from the CBCL is strongly correlated with gold-standard instruments of irritability measurement (Wilson, Cornacchio, Brotman, & Comer, 2021). Scores on these three items were summed to give a total irritability score at T1, T2 and T3, with each having a possible score of 0 – 6. We constructed our latent factor of early childhood irritability by specifying T1, T2 and T3 irritability scores as observed variables, and T1, T2 and T3 age of assessment as predictors of T1, T2 and T3 observed variables. The latent factor score therefore represents a weighted average of individual levels of irritability across the T1-T3 period which takes account of variability in the age of assessment across the timepoints. Confirmatory factor analyses to check all timepoints loaded in a comparable manner indicated that T1, T2 and T3 measurements all loaded significantly on our irritability factor (loadings = .72-.85; all *p*s < .001), but as the model was saturated, model fit indices were not available. Internal reliability was good (α = .86). Factor scores were extracted for each participant.

#### Peer Relationships

To derive an estimate of the quality of peer relationships at age 10 (T8), we used four items relevant to the construct, two derived from the Friendships Questionnaire used in the National Longitudinal Survey of Children and Youth (NLSCY; (Statistics Canada, 2007)) (“How many friends does s/he have?”, “During last six months, how well has s/he gotten along with other kids”, both rated on a 5-item Likert scale, with the infrequently selected scores of 5 recoded to a score of 4, possible score range 0 – 4), one from the Autism Diagnostic Interview-Revised (Rutter, Le Couteur, & Lord, 2003) (where parents rated the quality of their child’s friendships from *clear reciprocal friendship* (0) to *no peer relationships* (3), possible score range 0 – 3), and one from the CBCL (6-18 years version; Achenbach & Rescorla, 2001) (“Compared to other children their age, how well do they get along with other kids?”, rated *worse* (1), *the same* (2) or *better* (3), possible score range 1 – 3). Confirmatory factor analysis in Mplus using WLSMV for ordinal data suggested an additional correlation should be specified between the CBCL item and the Friendships Questionnaire item “During last six months, how well has s/he gotten along with other kids?” (using the modindices command). This was added; in the final model all indicators loaded significantly on the proposed factor (loadings = .26 - 1.00; all *p*s < .01) and model fit was good (χ^2^(1) =.03, *p* = .87, RMSEA = 0.00, TLI and CFI = 1.00). Internal consistency fell below the threshold of acceptability (α = .59). Factor scores were extracted for each participant.

#### Educational Engagement

Children’s level of engagement at school was rated by three items from the teacher-rated Adaptive Functioning subscale of the CBCL (6-18 years version; Achenbach & Rescorla, 2001). Teachers rated how hard the child was working, the appropriateness of a child’s behaviour and how much they were learning. Each item was rated on a 7-item Likert scale ranging from 1 (*Much Less*) to 7 (*Much Mor*e). The infrequently selected scores of 6 and 7 were recoded to 5, giving a possible score range of 1 – 5. As the model was saturated, model fit indices were not available, but all items loaded significantly on the proposed factor (loadings = .81 - .84; all *p*s < .001). Internal consistency was good (α = .86). Factor scores were extracted for each participant.

#### Childhood and Adolescent Depression Symptoms

Depression symptoms were measured upon study entry (T1) and at age 14 (T9) by the DSM-derived Depressive Problems subscale of the parent-rated CBCL (1.5-5/6-18 years version respectively; Achenbach & Rescorla, 2001). Items were rated as *not true* (0), *somewhat or sometimes true* (1), or *very true/often true* (2). In the CBCL 1.5-5 version the subscale consists of 10 items, giving a possible score range of 0 – 20, whereas in the 6-18 years version the subscale consists of 13 items, giving a possible score range of 0 – 26. Internal consistency was acceptable (α = .69 at T1, α = .77 at T9).

#### Covariates

The following variables, all measured at T1 (baseline), were included as covariates; sex assigned at birth, income (coded on an 11-point ordinal scale, 1 = <$5,000 CAD to 11 = >$80,000 CAD), marital status (single/married), siblings (present/absent), severity of maternal depression symptoms (as measured by the depression subscale of the Symptom Checklist-90-R, formed of 16 items with a possible score range 0 – 4; Derogatis, 1992), cognitive ability (as measured by the Merrill-Palmer-Revised Developmental Index Standard Score, possible score range 0 – 160; Roid & Sampers, 2004), autism symptoms (as measured by the ADOS - Calibrated Severity Score, possible score range 0 – 10; Lord et al., 2000) and site (with Montreal as the reference category). We included this combination of covariates as they have been associated with outcome variables of interest or similar constructs (e.g., conduct problems) in previous research (e.g., Costello et al., 2003; Eyre et al., 2017; Loeber & Stouthamer-Loeber, 1986; Mulkey, Crain & Harrington, 1992; Murray et al., 2010), or because there was some evidence they could impact associations between predictor, mediator and/or outcome (e.g., Humphreys et al., 2019; East & Rook, 1992).

### Statistical Analysis

Maternal and child depression scores were transformed using Box-Cox transformation. First, structural equation models tested longitudinal associations between early childhood irritability and age 14 depression symptoms, while accounting for baseline income, parental marital status, presence of siblings, maternal depression symptoms, cognitive ability, autism symptoms, site and age at assessment at the age 14 timepoint. We performed an additional sensitivity analysis to check if any observed associations could be explained by continuity in depression by re-running the analyses with baseline depression scores included as a covariate. Next, mediation models were constructed, specifying paths from early childhood irritability to both age 10 peer relationships and educational engagement and age 14 depressive symptoms, paths from age 10 peer relationships and educational engagement to age 14 depressive symptoms and a correlation between peer relationships and educational engagement. Income, maternal age at study entry, number of siblings, maternal depression symptoms, cognitive ability, autism symptoms, site, age at assessment at the age 10 timepoint and age at assessment at the age 14 timepoint were included as covariates. We calculate estimates of the indirect effect of peer relationships and educational engagement and associated percentile 95% confidence intervals (CIs) using bootstrapping with 200 repetitions. We conducted an additional sensitivity analysis where we re-ran models adjusting for early childhood ADHD symptoms due to the co-occurrence of irritability and ADHD, and the reported impact of ADHD on our mediators and outcome of interest (e.g., see Humphrey et al., 2013; Powell et al., 2020; Shaw, Stringaris, Nigg, & Leibenluft, 2014) (see Supplementary Materials for details on measurement of ADHD symptoms). We also re-ran mediation models using the single highest loading item on the peer relationships factor (“How many friends does s/he have?”) to check findings were not driven by measurement error in the peer relationships latent factor; results were unchanged (see Supplementary Materials). All longitudinal models were estimated in Stata 16 using full information maximum likelihood, which produces unbiased estimates if variables that predict missingness are included the model (e.g., in this case income and maternal age at study entry). We report both unstandardized (b) and standardized (β) coefficients.

## Results

### Associations between Early Childhood Irritability and Adolescent Depression

Early childhood irritability was significantly associated with depression symptoms at age 14 (b = .38, 95% CIs [.20, .56], *p* < .001, β = .31). Aside from maternal mental health (b = .87, 95% CIs [.15, 1.59], *p* = .02, β = .19), none of the other baseline covariates significantly predicted depression symptoms at 14 years (see Table 2 for details). In sensitivity analyses including baseline depression symptoms, the main effect of irritability remained significant (b = .21, 95% CIs [.03, .40], *p* = .03, β = .17) and baseline depression symptoms were positively associated with depression symptoms at 14 years (b = .39, 95% CIs [.20, .57], *p* < .001, β = .35).

### Mediation of Associations by Peer Relations and Educational Engagement

**Figure 1:**
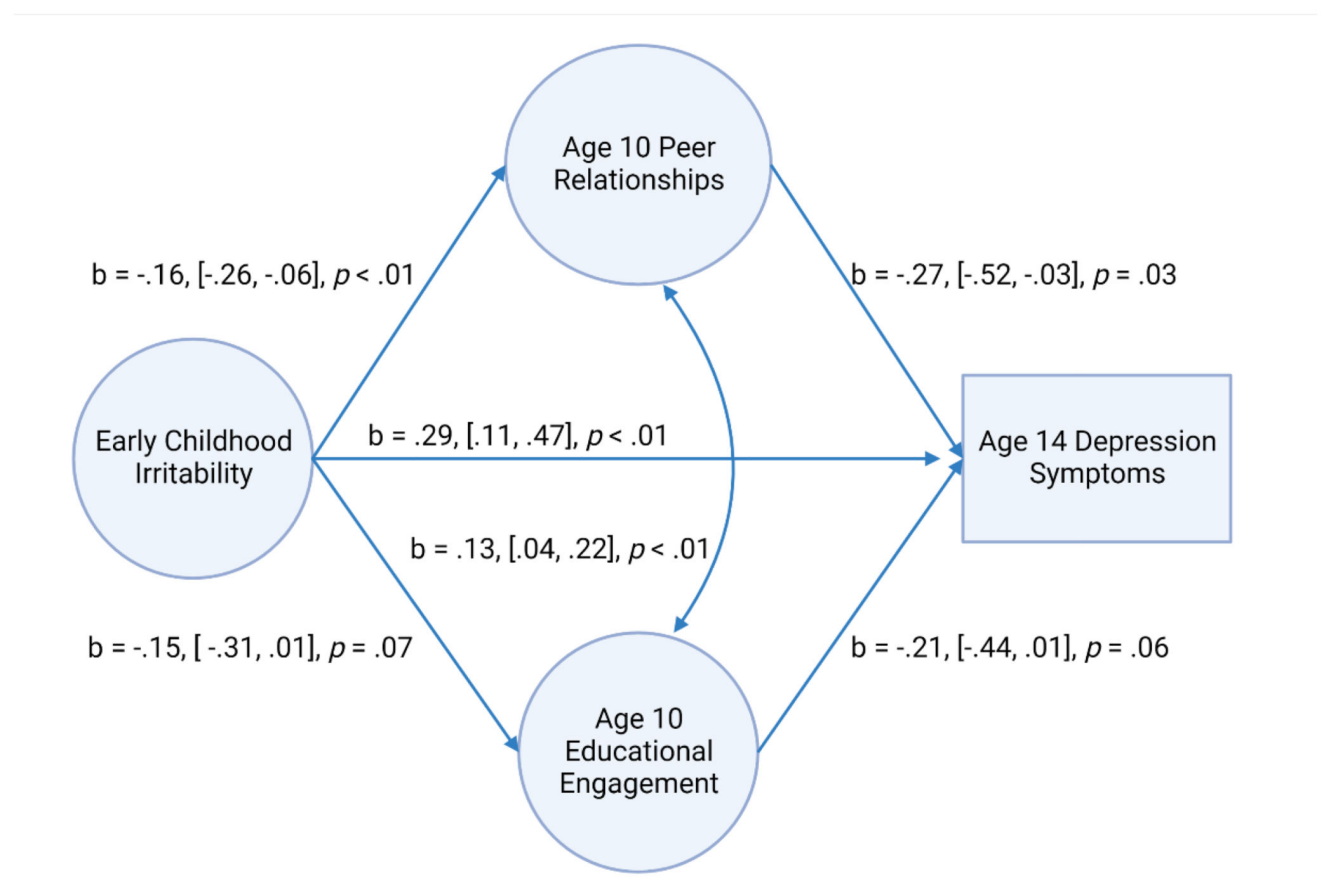
Longitudinal Pathways between Early Childhood Irritability and Adolescent Depression Symptoms in Autistic Youth. Created with BioRender.com. *Note: Square brackets indicate 95% confidence intervals*.

Higher irritability in early childhood was associated with lower peer relationship scores (b = - .16, 95% CIs [-.26, -.06], *p* < .01, β = -.21) (see Figure 1). All other covariate associations with peer relationships were non-significant (*ps* > .10). Early childhood irritability was not associated with education engagement at age 10 (b = -.15, 95% CIs [ -.31, .01], *p* = .07, β = - .15). Of all the covariate effects, baseline autism symptoms (b = -.13, 95% CIs [-.23, -.04], *p* < .01, β = -.25) were associated with lower educational engagement scores at age 10, and significant site effects were also found, such that both the Hamilton (b = -.45, 95% CIs [-.86, -.04), *p* = .03, β = -.17) and the Edmonton sites (b = -1.16, 95% CIs [-1.92, -.39), *p* < .01, β = -.44) had lower educational engagement scores compared to Montreal.

Lower peer relationship scores (b = -.27, 95% CIs [-.52, -.03], *p* = .03, β = -.17), but not educational engagement (b = -.21, 95% CIs [-.44, .01], *p* = .06, β = -.18), were associated with higher depression symptoms at age 14. Early childhood irritability (b = .29, 95% CIs [.11, .47], *p* < .01, β = .24) and baseline maternal depression (b = 1.01, 95% CIs [.29, 1.72], *p* < .01, β = .21) remained significant predictors of age 14 depression symptoms. Peer relationship scores and education engagement scores at age 10 were significantly correlated (b = .13, 95% CIs [.04, .22], *p* < .01, β = .25). Bootstrapped models found a significant indirect effect of peer relationships (b = .04, bootstrapped 95% CIs [.01, .12]), but not educational engagement (b = .03, bootstrapped 95% CIs [-.01, .11]). Comparison of the indirect and the total effects suggested around 13% of the observed association between childhood irritability and adolescent depression symptoms was mediated by difficulties in peer relationships.

### Sensitivity Analyses Adjusting for Early Childhood ADHD Symptoms

In models testing the association between early childhood irritability and adolescent depression symptoms with early childhood ADHD included as an addition covariate, irritability (b = .27, 95% CIs [.07, .48], β = .22, p < .01) and maternal depression symptoms (b = .87, 95% CIs [.16, 1.58], β = .18, p = .02) remained significant predictors of later depression. The coefficient of effect for ADHD was also at significance (b = .21, 95% CIs [.01, .42], β = .17, p = .05).

In mediation models, when early childhood ADHD was included as an addition covariate, the effect of irritability on peer relationships became non-significant (b = -.09, 95% CIs [-.21, .03]. β = .12, p = .14), and ADHD was a significant predictor of peer relationships (b = -.13, 95% CIs [-.25, .01], β = .17, p = .03). Early childhood ADHD was not a significant predictor of educational engagement (b = -.19, 95% CIs [-.39, .01], β =-.19, p = .06), and baseline autism symptoms (b = -.13, 95% CIs [-.22, -.04], β = -.24, p <.01) and the Hamilton site (b = -1.02, 95% CIs [-1.79, -.24], β = -.39, p = .01) remained significant predictors of educational engagement. The direct effect of irritability on adolescent depression remained significant (b = .24, 95% CIs [.04, .44], β =.20, p = .02), and there were no direct effects of ADHD symptoms on adolescent depression (b = .11, 95% CIs [-.10 .33], β = .09, p = .30). The indirect effect of peer relationships became non-significant (b = .02, bootstrapped 95% CIs [.02, .08]). The effect of educational engagement remained non-significant (b = .01, bootstrapped 95% CIs [-.01, .08]).

## Discussion

The current study reports on the long-term impact of early childhood irritability in autistic children in terms of peer relations, educational engagement and depressive symptoms. Similar to reports for non-autistic (i.e., typically developing) children, we found a positive association between early childhood irritability and depression symptoms at age 14, which remained significant when adjusting for the co-occurrence of irritability and depression symptoms in early childhood. Mediation analyses tested two mechanisms in middle childhood that could explain the association between irritability and later depression symptoms in adolescence. The first hypothesized mechanism, the impact of irritability on peer relationships, was supported, as indicated by a statistically significant indirect pathway from irritability to depression through a negative impact on peer relationships (although the percentage of variance explained was modest). The second hypothesized mechanism, the impact of irritability on educational engagement, was not supported.

The current results suggest that the well-documented association in typically developing populations between irritability and later depression (Vidal-Ribas et al., 2016; Vidal-Ribas & Stringaris, 2021) is also present in autistic individuals. We highlight that the current association was adjusted for parental marital status, income, presence of siblings, maternal depression, cognitive ability and autism symptoms (measured at the same time as irritability) and remained significant in analyses that adjusted for other co-occurring psychiatric symptoms (both depression and ADHD). In addition to the effect of early childhood irritability on adolescent depression, we also found a significant effect of maternal depression, measured at baseline when the children entered the study. There is evidence that such associations between maternal and child depression could reflect either an environmental path (McAdams et al., 2015), for example, where being parented by a mother who is experiencing depressive symptoms increases the likelihood the child themselves will experience depression, or a genetic path (Gjerde et al., 2021), such that the child is more likely to inherit genetic liability for depression from their parents, or interactions between environmental and genetic influences. Genetically informative designs (e.g., twin and adoption studies) in autistic samples are needed to tease apart the complex contributions of each aetiological pathway. It is important to note here, as in many other studies, mothers rated both their own and their children’s depression symptoms, which could lead to inflated estimates of effect due to shared method variance.

From a clinical perspective, the current finding of an association between early childhood irritability and adolescent depression symptoms emphasizes the importance of screening for and intervening on irritability in young autistic children, as this may decrease the likelihood of experiencing depression symptoms in adolescence, which is elevated in autistic populations (Oakley et al., 2021; Pezzimenti et al., 2019). There is evidence that both parent-focused (Tarver et al., 2019) and pharmacological (Fallah et al., 2019; Fung et al., 2016) interventions may be effective in reducing irritability specifically in autistic children. Additionally, some view irritability as one of many behavioural manifestations of emotion dysregulation (others being anxiety, self-injury, aggression; Mazefsky et al., 2013). Accordingly, a focus on identifying triggers of emotional response and strengthening regulatory abilities may also be beneficial (Moltrecht, Deighton, Patalay, & Edbrooke-Childs, 2021). Better understanding of the individual (e.g., cognitive profile, temperamental factors) and environmental (e.g., parenting styles, socio-economic status, exposure to other known risk factors for psychopathology) characteristics associated with high levels of irritability in autistic children will allow for the development of more precisely targeted interventions. One interesting line of research in terms of individual factors is the link between cognitive inflexibility, often noted in autistic youth, and irritability. There is some evidence to suggest an association between cognitive inflexibility and irritability in both non-autistic (Li et al., 2017) and autistic youth (Hollocks et al., 2021; Ozsivadjian et al., 2020; Simonoff et al., 2012), but whether these associations reflect a causal mechanism remains relatively unexplored. It may be that the strategies required to regulate negative emotional responses in part rely on cognitive flexibility (amongst other executive functions), such that this is one mechanism that partly explains the heightened prevalence of irritability in autistic populations.

Mediation analyses found a significant indirect effect of peer relationships, such that around 13% of the observed association between early childhood irritability and depression symptoms at age 14 could be explained by the knock-on effects of irritability on peer relationships at age 10. The association between irritability and peer relationships could reflect the impact that difficulties in the control of emotions and behaviours has upon the formation of social relationships. In turn, young people who have difficulties in peer relationships may experience feelings of rejection and failure, which in turn place them at higher risk for developing depression (i.e., the *failure model*; Burke et al., 2005; Patterson & Capaldi, 1990). We highlight that the direct pathway between early childhood irritability and adolescent depression remained significant in mediation models, suggesting that other unmeasured factors beyond peer relationships are also contributing to this association. Nonetheless, results suggest that a focus on supporting positive peer relationships in autistic children, especially those who exhibit irritability, may promote positive outcomes in adolescence. These could include helping autistic children to build social skills and confidence in friendships (Laugeson, Gantman, Kapp, Orenski, & Ellingsen, 2015) and supporting schools to implement classroom interventions focused on understanding of neurodiversity to promote inclusion (e.g., the Learning About Neurodiversity Project, https://dart.ed.ac.uk/research/leans/). Furthermore, recent studies using general population samples have reported the same mechanism may be present with childhood ADHD symptoms as the predictor of interest (Powell et al., 2020), suggesting that other characteristics that often co-occur with irritability (for which there are also evidence-based interventions) may also be important when thinking about the drivers of adolescent depression. Indeed, our additional sensitivity analyses found that whilst the association between early childhood irritability and depression remained significant when adjusting for co-occurring ADHD, the proposed indirect effects through peer relationships became non-significant, and thus may be in part driven by the co-occurrence of ADHD symptoms and irritability. Results from these sensitivity analyses require further investigation with more precise measures and multi-informant designs, as in the current analyses we were limited in our ability to determine specificity of effects by the fact that irritability and ADHD were measured by the same rater using the same instrument. Finally, we highlight that many studies focus on more extreme examples of negative peer relationships (e.g., bullying) as risk factors for internalizing symptoms in clinical samples of autistic children (Rodriguez, Drastal, & Hartley, 2021) and others have reported that peer victimization mediated associations between childhood irritability and suicidal behaviour in adolescence in typically developing children (Forte et al., 2021). Although it is possible that children who scored lower on our measures of peer relationships had also experienced bullying, our findings highlight the importance of the negative impacts of loneliness or friendship difficulties in increasing the risk of depression symptoms in autistic adolescents. Finally, we note that inspection of the loadings for our peer relationships factor suggests that this largely measured the number of friendships. Whether similar results are obtained with more precise measurement of the quality of children’s peer relationships (which might be harder for parents to report) should be tested in future work. More precise measures will likely have higher reliability, which is a limitation of our current measure of peer relationship quality.

We did not find any evidence that educational engagement mediated the association between early childhood irritability and adolescent depression symptoms. In general population samples, lower educational achievement mediates the association between childhood ADHD behaviours and adolescent depression (Powell et al., 2020). However, studies investigating the replicability of the association between academic failure and peer relationship difficulties on later depression in samples of children at risk for behaviour problems find less consistent results for academic failure (Patterson & Stoolmiller, 1991). This may be because academic failure is less potent a risk factor for later depression, or because there is more variation in how academic failure is measured compared to peer difficulties, or because different studies used samples consisting of different groups of children, all which impact the replicability of results. Finally, it should also be held in mind that our measurement of educational engagement was rated by teachers, whereas all other measures were rated by parents. Additionally, in the current study, we measured educational engagement rather than educational *attainment*. This was largely due to the multi-site design of Pathways, in which educational systems differ substantially within and across regions, combined with the fact that children who took part completed both mainstream and adapted/individualized programs, making calibration of school grades across the total sample challenging. Instead, we used teachers’ reporting on how hard the child was working, the appropriateness of a child’s behaviour, and how much they were learning. Although these constructs are correlated with grade attainment (see Valiente et al., 2008), actually having lower grades may be what contributes to risk for later depression through lowered self-confidence (as reported elsewhere; Vaillancourt et al., 2013), rather than how engaged the child is at school. Indeed, work from the ADHD field has found mediation effects when using examination results as a measure of educational achievement when looking at the impact of ADHD symptoms in general-population cohorts (Powell et al., 2020), but not when using academic stress/functioning as the mediator, either in cross-sectional samples of children with and without ADHD, or longitudinal samples of children at risk for depression (Humphrey et al., 2013). An alternative interpretation of the lack of mediation effects is that this mechanism is not operating in autistic children. In the current sample, all children had a formal diagnosis of autism. It may be that educator and parent knowledge of differences in learning style may have meant less weight was placed on grade achievement and more on well-being and school participation (especially for children in specialist classes). Thus, children may have felt less pressure and peer comparison with regard to grade attainment, lessening potential negative impacts. Studies that examine the impact of grade attainment on mental health in autistic as compared to typically developing children are required to test this working hypothesis. Additionally, in the current model we included early childhood cognitive ability and autism symptomatology as covariates, but they may instead function as moderators, especially with regard to pathways between educational attainment and depression outcomes. This should be tested in future studies.

Key strengths of this study include a relatively large longitudinal cohort of autistic children who have been followed from early childhood to adolescence, with both parent- and teacher-rated measures. The main limitation of the current work is the reliance on parent-report for all measures apart from educational engagement, which was rated by teachers. This means that shared methods variance may have contributed to results. Although self-report is typically considered preferable when assessing internalizing symptoms, such as those characteristic of depression, this may be difficult for autistic adolescents, who are more likely to have difficulties with the identification and communication of internal states (Bird & Cook, 2013). More work is needed to better understand what contributes to agreement and discrepancies between self- and other-rated measures in autistic populations, and whether existing measures of internalizing symptoms are suitable for autistic children and adolescents, including those with intellectual and/or communicative disabilities (e.g., Kaat & Lecavalier, 2015; Wigham & McConachie, 2014). We also highlight that our measure of irritability was taken from the CBCL, which was not designed specifically to measure this construct (unlike other measures, for example the Affective Reactivity Index; Stringaris et al., 2012b), although recent work suggests it correlates strongly with these more precise instruments (Wilson, Cornacchio, Brotman, & Comer, 2021). Similarly, our measure of peer relationships was not taken from a validated questionnaire as no such measure was available at the time of data collection; more recent validated measures (e.g., Toomey et al., 2016) should be used to confirm the replicability of our results. It should also be acknowledged that measuring mediators at an earlier time point than depressive symptoms does not guarantee that these factors precede depressive symptoms; symptom-driven pathways (i.e., from internalizing symptoms to educational outcomes and peer victimization) have been reported elsewhere (Saint-Georges & Vaillancourt, 2020; Vaillancourt et al., 2013). Future work should combine repeated measurements with appropriate statistical models to demarcate the directionality of effects between the three domains (Hamaker, Kuiper, & Grasman, 2015). Additionally, future directions could include how to better account for time-varying confounders, as some variables measured at T1 (e.g., parental mental health, autism symptoms) likely vary between early childhood and adolescence. Future work is also needed to examine whether similar effects are found in autistic individuals who receive a diagnosis later in life, as participants in the current sample had to have received a clinical diagnosis of autism relatively early in development to enter the study, and thus may only represent one subpopulation of autistic people. Attrition analyses also suggested that within the Pathways cohort, families with lower incomes and younger mothers on study entry were more likely to drop out of the study over time. This highlights potential sample biases that should be held in mind when extrapolating current results to autistic youth more broadly. Although our analytic method adjusted for the impact of these particular variables on model estimates, there are likely other factors that predict missingness in this cohort that were not captured and thus could be causing unmeasured bias. From a theoretical point of view, we also highlight that without genetic information we cannot rule out pleiotropy. Indeed, in general population samples there is evidence for overlap in the genetic factors that influence irritability and depression (Stringaris et al., 2012a), which could in part account for phenotypic associations between the two constructs. Incorporating mediation analyses within randomized control trials targeting pre-school irritability would be one way to test causality.

Current results suggest that higher levels of irritability in young autistic children are associated with greater depression symptoms in adolescence, and that the knock-on effects of irritability on peer relationships may explain the association between the two (although ADHD symptoms may also play a role). Results have significant clinical implications as they highlight the importance of screening and intervention for co-occurring irritability and supporting positive peer relationships in order to promote positive long-term outcomes for autistic children.

## Supplementary Material

Supplementary Materials

## Figures and Tables

**Table 1:** Descriptive Statistics for Included Sample

	N	Mean	Standard Deviation	Min-Max	N (%) scoring each category	
**Preschool**						
T1 CBCL irritability	362	2.38	1.68	0 – 6		
T1 Age of Assessment	365	3.41	.76	1.98 – 5.28		
T2 CBCL irritability	321	2.09	1.73	0 – 6		
T2 Age of Assessment	327	3.99	.79	2.46 – 6.01		
T3 CBCL irritability	297	1.93	1.70	0 – 6		
T3 Age of Assessment	301	4.51	.76	3.01 – 6.38		
T1 M-P cognitive standard score	321	57.51	26.10	10 – 136		
T1 ADOS-CSS autism symptoms	385	7.60	1.72	2 – 10		
T1 CBCL DSM depression symptoms	364	3.98	3.07	0 – 16		
T1 Symptom Checklist-90-R maternal depression	346	.96	.83	0 – 4		
T1 Sex	390				Male Female	331 (85%) 59 (15%)
T1 Siblings	390				Absent Present	130 (33%) 260 (67%)
T1 Parental income	363	Median= < $70,000			Lower quartile Upper quartile	<$40,000 >$80,000
T1 Parental marital status	372				Single Married	43 (12%) 329 (88%)
T1 Maternal Age at Study Entry	389	35.19	5.48	20.74 – 51.09		
T1 Site	390				Halifax Montreal Hamilton Vancouver Edmonton	53 (14%) 130 (33%) 60 (15%) 90 (23%) 57 (15%)
**Time 8 (Age 10)**						
T8 Age of Assessment	177	10.76	.24	10.14 – 11.90		
RQ Number of Friends	178				None 1 2 to 3 4 or more	58 (33%) 28 (16%) 75 (42%) 17 (10%)
RQ Gets Along with Other Children	168				Not Too Well Pretty Well Quite Well Very Well	12 (7%) 51 (30%) 62 (37%) 43 (25%)
ADI Current Friendships	199				0 1 2 3	50 (25%) 72 (36%) 44 (22%) 33 (17%)
CBCL Gets Along with Other Children	170				Worse Average Better	50 (29%) 104 (61%) 16 (9%)
CBCL-Teacher Working Hard	142	2.99	1.44	1 – 5		
CBCL-Teacher Behaving	142	2.95	1.44	1 – 5		
CBCL-Teacher Learning	142	2.88	1.43	1 – 5		
**Time 9 (Age 14)**						
T9 Age of Assessment	167	13.84	.83	12.02 – 15.38		
CBCL DSM depression symptoms	165	3.84	3.52	0 – 14		

ADOS -CSS = Autism Diagnostic Observation Schedule – calibrated severity score; CBCL = Child Behavior Checklist, MP = Merrill-Palmer Note: Numbers for age of assessment and subscale scales for the CBCL may differ within timepoint due to questionnaire non-completion

**Table 2:** Early Childhood Predictors of Adolescent Depression Symptoms in Autistic Children

Predictor	b	95% CIs		β	p value
		Lower Bound	Upper Bound		
**Early childhood irritability**	**.38**	**.20**	**.56**	**.31**	**<.001**
T1 sex	.31	-.12	.74	.10	.16
T1 income	-.01	-.08	.07	-.01	.95
T1 marital status	.28	-.40	.95	.08	.42
T1 maternal age at study entry	-.01	-.04	.02	-.07	.37
T1 siblings	-.31	-.63	.01	-.13	.06
**T1 maternal depression**	**.87**	**.15**	**1.59**	**.19**	**.02**
T1 cognitive ability	.01	-.01	.01	.10	.18
T1 autism symptoms	-.03	-.11	.06	-.04	.58
Site – Halifax	-.29	-.80	.25	-.09	.30
Site – Hamilton	.10	-.37	.58	.03	.67
Site – Vancouver	.07	-.35	.49	.03	.75
Site - Edmonton	.20	-.47	.87	.07	.55
T9 age of assessment	.07	-.20	.34	.05	.62
Sensitivity Analyses Including T1 Depression Symptoms					
**Early childhood irritability**	**.21**	**.03**	**.40**	**.17**	**.03**
**T1 depression symptoms**	**.39**	**.20**	**.57**	**.35**	**<.001**
T1 sex	.29	-.12	.70	.10	.17
T1 income	-.01	-.08	.06	-.02	.79
T1 marital status	.35	-.29	.99	.11	.28
T1 maternal age at study entry	-.01	-.03	.03	-.01	.92
T1 siblings	-.27	-.57	.04	-.11	.09
T1 maternal depression	.65	-.04	1.34	.14	.07
T1 cognitive ability	.01	-.01	.01	.11	.15
T1 autism symptoms	-.03	-.12	.05	-.05	.45
Site – Halifax	-.39	-.90	.11	-.12	.13
Site – Hamilton	-.01	-.47	.45	-.01	.96
Site – Vancouver	-.08	-.48	.33	-.03	.71
Site - Edmonton	.03	-.61	.67	.01	.93
T9 age of assessment	.07	-.19	.33	.05	.59

Note: Early childhood irritability was measured across T1-T3; T1; mean age 3.41 years, T2; mean age 3.99, T3; mean age 4.51 years, T9; mean age 13.84 years.
