# Supplementary material for: Longitudinal Associations between Early Childhood Irritability and Adolescent Depression Symptoms in Autistic Children are Mediated by Peer Relationships but Not Educational Engagement
Source: Dev Psychopathol. Author manuscript; Available in PMC 2024 Feb 22. (PMC7615659; doi:10.1017/S0954579422001316)
Supplement: Supplementary Materials [file EMS157074-supplement-Supplementary_Materials.pdf]

Supplementary Materials

Measurement of Childhood ADHD Symptoms

Childhood ADHD symptoms were measured at T1, T2 and T3 using the Attention Deficit/Hyperactivity Problems subscale of the parent-report CBCL (1.5-5 years version) (Achenbach & Rescorla, 2001). We constructed our latent factor of early childhood ADHD symptoms by specifying T1, T2 and T3 ADHD symptom scores as observed variables, and T1, T2 and T3 age of assessment as predictors of T1, T2 and T3 observed variables. The latent factor score therefore represents a weighted average of individual levels of ADHD symptoms across the T1-T3 period which takes account of variability in the age of assessment across the timepoints. Confirmatory factor analyses to check all timepoints loaded in a comparable manner indicated that T1, T2 and T3 measurements all loaded significantly on our irritability factor (loadings = .68-.85; all *p*s < .001), but as the model was saturated, model fit indices were not available. Internal reliability was good (α = .82). Factor scores were extracted for each participant.

Additional Analyses of Peer Mediation Effects

To check that poor measurement in our latent factor of peer relationships was not driving our findings, we re-ran the mediation models using the item that loaded highest on the peer relationships factor score ("How many friends does s/he have?", derived from the Friendships Questionnaire used in the National Longitudinal Survey of Children and Youth; Statistics Canada, 2007) as the mediator. We find the coefficient for the indirect effect remains comparable with this single item measure (b=.04, 95% bootstrapped CIs [.01, .11].

References

Achenbach, T. M., & Rescorla, L. (2001). *Manual for the ASEBA school-age forms & profiles: An integrated system of multi-informant assessment*: Aseba: Burlington, VT.

Statistics Canada. (2007). Microdata user guide. National Longitudinal Survey of Children and Youth. Retrieved from https://crdcn.org/datasets/nlscy-national-longitudinal-survey-children-and-youth
